# THE EFFECT OF DISRUPTIVE MEDICAL EVENTS ON MORTALITY IN PEOPLE WITH AND WITHOUT DEMENTIA

**DOI:** 10.1093/geroni/igac059.1625

**Published:** 2022-12-20

**Authors:** Lauren Hunt, Sean Morrison, Siqi Gan, Edie Espejo, W John Boscardin, Rebecca Rodin, Katherine Ornstein, Alexander Smith

**Affiliations:** University of California, San Francisco, San Francisco, California, United States; Icahn School of Medicine at Mount Sinai, New York, New York, United States; University of California, San Francisco, San Francisco, California, United States; University of California, San Francisco, San Francisco, California, United States; University of California, San Francisco, San Francisco, California, United States; Icahn School of Medicine at Mount Sinai, New York, New York, United States; Johns Hopkins University, Baltimore, Maryland, United States; University of California, San Francisco, San Francisco, California, United States

## Abstract

Disruptive medical events such as pneumonia and hip fracture occur more frequently among older adults with dementia than those without dementia. It is not well-understood whether these events increase the risk of mortality to a greater extent for people with dementia (PWD) compared to people without dementia (PWoD). Using data from the Health and Retirement Study linked to Medicare claims, we estimated the impact of hip fracture and pneumonia on risk of mortality among 700 PWD and 12,438 PWoD using a Cox proportional hazards model. PWD had a higher risk of mortality both in the case of hip fracture (HR 1.64, 95% CI 1.31, 1.96) and pneumonia (HR 1.21 95% CI 1.09, 1.34) compared to PWoD who experienced those events. This study provides evidence that dementia may increase mortality after a disruptive medical event and suggests that the clinical course of dementia may not always be slow and gradual.

